# Cardiac Magnetic Resonance Relaxometry Parameters, Late Gadolinium Enhancement, and Feature-Tracking Myocardial Longitudinal Strain in Patients Recovered from COVID-19

**DOI:** 10.3390/jcdd10070278

**Published:** 2023-06-29

**Authors:** Jadwiga Fijalkowska, Anna Glinska, Marcin Fijalkowski, Katarzyna Sienkiewicz, Dorota Kulawiak-Galaska, Edyta Szurowska, Joanna Pienkowska, Karolina Dorniak

**Affiliations:** 1 Second Department of Radiology, Medical University of Gdansk, 80-210 Gdansk, Poland; jfijalkowska@gumed.edu.pl (J.F.);; 2 First Department of Cardiology, Medical University of Gdansk, 80-210 Gdansk, Poland; 3 Department of Radiology, Medical University of Gdansk, 80-210 Gdansk, Poland; 4 Department of Noninvasive Cardiac Diagnostics, Medical University of Gdansk, 80-211 Gdansk, Poland

**Keywords:** non-ischemic cardiac injury, cardiac magnetic resonance imaging, coronavirus disease, late gadolinium enhancement, myocarditis, myocardial longitudinal strain

## Abstract

COVID-19 infection is associated with myocarditis, and cardiovascular magnetic resonance (CMR) is the reference non-invasive imaging modality for myocardial tissue characterization. Quantitative CMR techniques, such as feature tracking (FT) and left ventricular global longitudinal strain (GLS) analysis, have been introduced as promising diagnostic tools to improve the diagnostic accuracy of suspected myocarditis. The aim of this study was to analyze the left ventricular global longitudinal strain (GLS) and the influence of T1 and T2 relaxation times, ECV, and LGE appearance on GLS parameters in a multiparametric imaging protocol in patients who recovered from COVID-19. The 86 consecutive patients enrolled in the study had all recovered from mild or moderate COVID-19 infections; none required hospitalization. Their persistent symptoms and suspected myocarditis led to cardiac magnetic resonance imaging within 3 months of the diagnosis of the SARS-CoV-2 infection. Results: Patients with GLS less negative than −15% had significantly lower LVEF (53.6% ± 8.9 vs. 61.6% ± 4.8; <0.001) and were significantly more likely to have prolonged T1 (28.6% vs. 7.5%; *p* = 0.019). Left ventricular GLS correlated significantly with T1 (r = 0.303; *p* = 0.006) and LVEF (r = −0.732; *p* < 0.001). Left ventricular GLS less negative than −15% was 7.5 times more likely in patients with prolonged T1 (HR 7.62; 95% CI 1.25–46.64). The reduced basal inferolateral longitudinal strain had a significant impact on the global left ventricular longitudinal strain. ROC results suggested that a GLS of 14.5% predicted prolonged T1 relaxation time with the best sensitivity and specificity. Conclusions: CMR abnormalities, including a myocarditis pattern, are common in patients who have recovered from COVID-19. The CMR feature-tracking left ventricular GLS is related to T1 relaxation time and may serve as a novel parameter to detect global and regional myocardial injury and dysfunction in patients with suspected myocardial involvement after recovery from COVID-19.

## 1. Introduction

COVID-19-associated myocardial injury is common and may occur as a direct result of myocardial viral infection or indirectly as a result of systemic inflammation, endothelial dysfunction, or microvascular thrombosis [1]. Previous studies have shown that myocardial involvement is associated with a worse prognosis in patients with COVID-19 [2,3]. When myocarditis is suspected, cardiovascular magnetic resonance (CMR) is the key non-invasive diagnostic tool [4]. Because of its exceptional combination of morphological and functional assessment with myocardial tissue characterization, CMR is an optimal imaging modality for detecting the typical signs of acute myocardial inflammation, such as contraction abnormalities, edema, hyperemia, and fibrosis. Although the current “Lake Louise Criteria” (LLC), [4] recently updated to include contemporary parametric techniques [5] for CMR-based diagnosis of myocarditis, have been well evaluated, additional parameters could improve the accuracy and precision of the diagnosis [6,7].

Wall motion assessment is paramount for the detection of contractile functional impairment. With good endocardial border delineation and high tissue contrast, CMR offers significant advantages in this regard. However, given subjectivity and operator dependence, the need for quantification seems self-explanatory. Quantitation of myocardial deformation provides a more sensitive and robust evaluation of both atrial and ventricular function and has recently been a fast-advancing field of CMR, allowing for the detection of subclinical myocardial disease in a variety of cardiovascular conditions. Recent clinical studies have shown that global longitudinal strain (GLS) is a better diagnostic tool than left ventricular ejection fraction (LVEF) for many cardiac abnormalities, especially for detecting mildly impaired left ventricular function, allowing for early diagnosis [8]. These studies also reported a significant decrease in GLS without overt impairment of global LV systolic function. Strain analysis by echocardiography, including GLS, has been shown to predict total cardiovascular mortality more accurately than abnormalities in LVEF [9]. In addition, reduced GLS correlates with histologic findings of myocardial fibrosis, even in the absence of significant LVEF impairment, in the early stages of the disease [10]. Several techniques for CMR strain assessment have been proposed, but most (such as myocardial tagging [MT], displacement encoding [DENSE], and strain encoding [SENC]) require dedicated additional sequences and therefore are unlikely to be widely adopted in the nearest future. Recently, new quantitative CMR methods, such as feature tracking (FT)-based strain analysis, have been presented as powerful imaging tools to improve the diagnosis of myocarditis [11]. This parameter can be obtained and computed by many widely available CMR software brands and it utilizes the most basic widely available cine images, making this type of advanced analysis essentially possible at any cardiac-enabled MR scanner [12]. The parameters for CMR feature tracking myocardial strain are related to myocardial deformation and, in parallel to speckle tracking strain analysis by echocardiography, are believed to be a more sensitive parameter of functional abnormalities than LVEF [13]. Recent studies have suggested that clinicians seeking a more accurate diagnosis of myocarditis should consider a multi-parametric imaging protocol that combines several of the modern quantitative parameters [14].

To our knowledge, few studies have attempted to evaluate the relationship between myocardial longitudinal strain and other MRI parameters, especially in patients who have recovered from COVID-19. The aim of the present study was to analyze FT-derived left ventricular global longitudinal strain (GLS) and the influence of T1 and T2 mapping, ECV, and LGE extent on GLS parameters in a multiparametric imaging protocol in patients after recovery from COVID-19.

## 2. Materials and Methods

### 2.1. Study Population

A total of 86 consecutive patients who met all inclusion criteria and had no exclusion criteria were enrolled in the study at the post-COVID-19 cardiology outpatient clinic. The study’s inclusion criteria were as follows: 1. SARS-CoV-2 infection confirmed by reverse transcription-polymerase chain reaction (RT-PCR) swab; 2. Absence of clinical symptoms of active COVID-19 pneumonia; 3. Persistent symptoms, including chest pain, arrhythmia, dyspnea, or fatigue, suggestive of cardiac involvement. Exclusion criteria were a history of cardiac disease (except arterial hypertension) and general contraindications to contrast-enhanced CMR (including end-stage chronic kidney disease and severe claustrophobia). Patients with other known cardiac diseases were also excluded to minimize the effect of non-COVID-19-related diseases on CMR results. All subjects had recovered from COVID-19, and the course of the infection was mild or moderate; none of the patients required hospitalization. Due to persistent symptoms and suspected myocarditis, CMR was performed, with a median time between the scheduled CMR evaluation and the initial COVID-19 disease diagnosis of 10 (6–14) weeks.

The study was designed and conducted in accordance with the Declaration of Helsinki, and its protocol was approved by the Independent Bioethics Committee for Scientific Research at the Medical University of Gdansk (Approval No. NKBBN/475/2021). Written informed consent was obtained from all study participants.

### 2.2. CMR Image Acquisition and Analysis

Details of CMR acquisition and analysis have been described previously [3,5]. All participants underwent CMR examination on a 1.5-T scanner (Magnetom Aera or Magnetom Sola, Siemens Healthineers, Erlangen, Germany) with an 18-element phased-array cardiac coil, using the standardized imaging protocols described previously [3,5]. These included long-axis and short-axis cine series for anatomy and functional assessment, followed by cardiac parametric mapping sequences for longitudinal (T1) and transverse (T2) relaxation time measurements (MOLLI (Modified Look-Locker) sequence for T1 and a T2-prepared bSSFP sequence for T2 measurement; MyoMaps, Siemens Healthineers, Erlangen, Germany), as well as routine LGE assessment in the standard long axes and a short-axis stack using both fast single-shot bSSFP inversion recovery and segmented phase-sensitive inversion recovery sequences, performed within 7–15 min after injection of 0.1 mmol/kg gadobutrol (Gadovist, Bayer AG, Leverkusen, Germany) [15]. The left ventricle was divided into 16 segments according to the American Heart Association (AHA) [16,17].

The GLS of the left ventricle was measured using CMR feature tracking, which involves the detection of “patterns of features” or “irregularities” in the endocardial border that are tracked and followed in successive frames of routine CMR cine [18]. Quantitative data on the deformation of the longitudinal orientation of the left ventricular myocardium were then analyzed using the Circle CVI software (Circle Cardiovascular Imaging, Calgary, AB, Canada) [19]. As the universal feature tracking strain reference values were not published to date, the GLS value was defined as significantly reduced when it was less negative than a threshold of −15%, which represents the lower limit (-2SD) of the reference range in well-established echocardiographic strain assessment [20]. To the best of our knowledge, the reported cut-off values for unequivocally abnormal FT longitudinal strain were variable, depending on methodology, cohort, and the presence or absence of a control group within these studies [21,22]. The cutoff of −15% seemed justified as, on one hand, it corresponds to the echocardiography cut-off and several authors reported FT-GLS values within a similar range [23], and on the other hand, it seems away from the potential grey zone for CMR-FT GLS, which has well-reported reference values in a meta-analysis of over 650 healthy individuals [12]. The presence of fibrosis by LGE was assessed visually and further categorized into epicardial, mid-wall, or diffuse/transmural patterns.

Diffuse fibrosis was assessed by extracellular volume calculations. Edema was determined by the signal intensity (SI) ratio of the myocardium to the skeletal muscle SI on T2-weighted images. All analyses, including traditional CMR features, were performed by the same two physicians (a cardiologist and a radiologist, with 5 and 11 years of experience in CMR, respectively, blinded to patient baseline characteristics and outcomes) using commercial software (SyngoVia VB40, Siemens Healthineers, Erlangen, Germany). In the case of discordant results, a consensus decision was reached with a third experienced CMR reader. In a second step, CMR-FT was performed independently, with the readers further blinded to all other CMR findings, including LGE and LVEF.

### 2.3. Statistical Analysis

Continuous data are presented as the mean and standard deviation (SD). Categorical data were presented as a percentage of the total. The normal distribution was tested using the Kolmogorov-Smirnov test. Continuous data from two groups were compared using the Student’s *t*-test or Mann-Whitney U test, depending on the distribution type. Categorical data were compared using the chi-squared test and Fisher’s exact test. Correlations were assessed with the Pearson or Spearman correlation test, depending on the distribution of the variables. We searched for independent risk factors for a diagnosis of global strain less negative than −15% using univariate logistic regression and then analyzed variables with *p* < 0.10 using multivariate logistic regression. Receiver operating characteristic (ROC) analysis was used to evaluate the predictive value of global T1 for global strain less negative than −15% and to determine the best cutoff for global T1 using the Youden index (*J*) method. A *p* value less than 0.05 was considered statistically significant. Data were analyzed using SPSS software v. 21 (IBM, Armonk, NY, USA).

## 3. Results

Finally, 81 patients (aged 42 ± 11 yrs; 59 [73%] females) were included in the study; 5 patients were excluded because the quality of their CMR images did not allow CMR feature tracking strain analysis due to artifacts. 

The baseline characteristics of the included patients are presented in Table 1.

**Figure 1:**
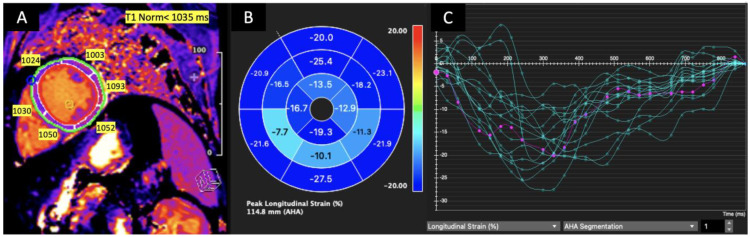
T1 relaxation time mapping for LV mid-segments in a mid-ventricular short axis slice (**A**), left ventricular peak longitudinal strain values bull’s eye (**B**). The strain curve graph shows the longitudinal strain of each LV segment versus time (green curves). The average peak longitudinal strain (violet curve) is decreased, and T1 is prolonged in the mid-inferior, mid-inferoseptal and mid-inferolateral segments in the patient with acute myocarditis. (**C**) Green for LV segments, violet or purple for average.

The mean GLS was −16% ± (−3), mean T1 was 1005 ± 34 ms mean T2 was 47.7 ± 3.7 ms, and mean ECV was 27± 5. Prolonged T1 was noted in 12 (15%) patients, prolonged T2 in 21 (26%) patients, and higher ECV in 13 (16%) patients. LGE was present in 52 (64%) patients. Overall, 7 (8.5%) patients met the LLC criteria for active myocarditis. Representative examples of longitudinal strain curves and LGE images from a patient with acute myocarditis are shown in Figure 1.

Patients with reduced left ventricular global longitudinal strain (GLS) had significantly lower LV ejection fraction, significantly more prolonged T1, and significantly more positive late gadolinium enhancement (Table 2).

Left ventricular global longitudinal strain correlates positively and significantly with T1 and negatively and significantly with LVEF (Table 3). There were no statistically significant correlations between LVEF and T1, T2 and ECV, where r = −0.38 (*p* = 073), r = −0.059 (*p* = 0.60), and r = −0.026 (*p* = 0.82), respectively. 

Multivariate logistic regression analysis revealed that, among the selected variables, prolonged T1 was a significant independent predictive factor for an abnormal GLS less negative than −15% (HR 7.62; 95% CI 1.23–46.64) (Table 4).

Univariate and then multivariate logistic regression were performed on CMR parameters, including segmental longitudinal strain and the continuous variables T1, T2, and ECV, for each of the 16 LV segments to evaluate the effect on reducing global LV longitudinal strain (GLS). Both longitudinal strain and T1 of three inferolateral LV segments (numbers 5, 6, and 10) were significantly affected in patients with reduced GLS in univariate logistic regression. However, only longitudinal strain for segment number 5 was significantly reduced in patients with reduced GLS in multivariate logistic regression (Table 5).

**Figure 2:**
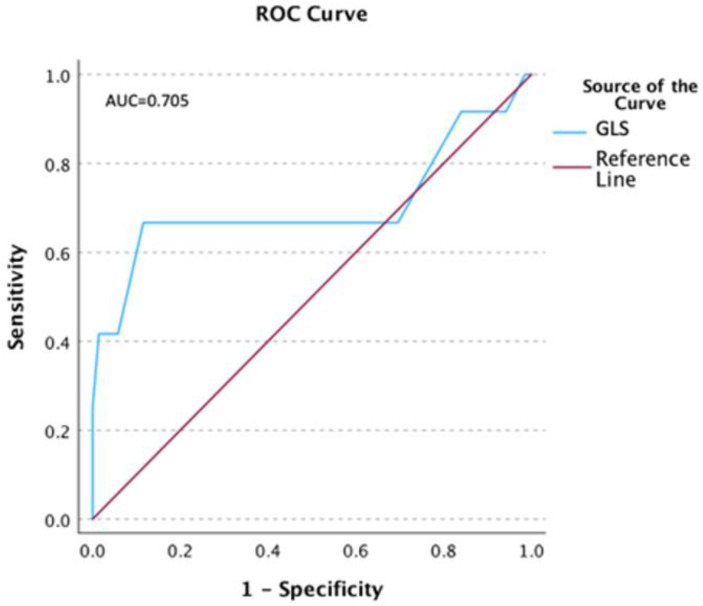
ROC for the T1 relaxation time and global longitudinal strain (GLS).

The ROC analysis was performed, and the results suggested that GLS equal to 14.5% predicts prolonged T1 relaxation time with the best combination of sensitivity (0.67) and 1-specificity (0.12), and borderline significance (*p* = 0.06) (Figure 2).

## 4. Discussion

In this study, we evaluated the global longitudinal strain (GLS) of the left ventricle derived from feature tracking and the effect of T1 and T2 mapping, ECV, and LGE on GLS in patients who had recovered from COVID-19. 

The main findings of this study are as follows:

1. CMR abnormalities, including myocarditis patterns, are common in patients who have recovered from COVID-19 and presented with protracted symptoms that could have resulted from cardiac involvement.
2. Patients with GLS less negative than −15% had significantly lower LVEF and a significantly higher number of segments with prolonged T1.
3. Left ventricular GLS was significantly positively correlated with T1 and significantly negatively correlated with LVEF.
4. Left ventricular GLS less negative than −15% was 7.5 times more likely in patients with prolonged T1.
5. Reduced longitudinal strain in the basal inferolateral segment had a significant effect on the global left ventricular longitudinal strain.
6. ROC results suggested that a GLS of −14.5% predicted prolonged T1 relaxation time with the best combination of sensitivity and specificity.

In our previous study, we evaluated the myocardial injury detected by TTE and CMR in healthcare workers recovering from non-severe COVID-19 [6]. Left ventricular ejection fraction was reduced in 29% of these cases as assessed by both TTE and CMR, and global LV longitudinal strain as assessed by echo was also slightly reduced in 39% of the subjects. Puntmann et al. demonstrated cardiac involvement by CMR in 78% of patients who recovered from COVID-19, where the majority of patients were asymptomatic or had at most mild symptoms. The results also indicated an increased likelihood of subsequent myocardial injury in both symptomatic and asymptomatic coronavirus infections and the possibility of cardiac damage. Of note, the outcomes were not related to the presence of symptoms of SARS-CoV-2 infection [24]. Further, CMR research revealed the possibility of persistent cardiac involvement in patients who have recovered from COVID-19. In fact, myocardial edema or fibrosis was observed in 58% of patients who had recently recovered from COVID-19 [25]. Another study by Xie et al. found that patients with COVID-19 infection have a high risk of cardiovascular involvement, including pericarditis and myocarditis, more than 30 days after infection, regardless of the need for hospitalization [26]. The prevalence of CMR abnormalities within 3 months of infection in the present study was similar to the previously analyzed studies.

The efficacy of regional left ventricular systolic dysfunction assessed by longitudinal strain in echocardiography is well established in patients with myocarditis. Leitman et al. demonstrated a high correlation between regional longitudinal strain and LGE detection in the same left ventricular regions in patients with myocarditis [27]. The investigators also showed that the locations of regional wall motion abnormalities and inflammatory features were similar for both STE and CMR and were mostly found in the lateral, inferior, and posterior wall segments. In the present study, the inferior and posterolateral segments of the left ventricle were also most commonly affected by infection. Similar results were shown by Kostakou et al. [28], who demonstrated that patients with acute myocardial infection had significantly reduced global longitudinal left ventricular strain assessed by echocardiography, regardless of normal LV ejection fraction. Furthermore, the presence of regional LV contractile abnormalities assessed by echocardiographic longitudinal strain was consistent with LGE findings in the same LV segments. This demonstrated that reduced segmental longitudinal strain in the inferolateral segments of the LV has a particularly high sensitivity and specificity for the diagnosis of myocarditis. It was also found that the reduced longitudinal strain of segment 5 had a significant effect on the reduction of global LV deformation. It is worth noting, that location of the reduced regional longitudinal strain in post-COVID-19 cohorts seems largely non-specific considering the recent literature [29,30]. In a study by Gao et al. including 47 patients with non-COVID-19 myocarditis and 39 healthy controls, myocardial injury detected by reduced myocardial strain was most commonly found in the inferior and inferolateral segments, which is consistent with our results [30]. In contrast, in patients with non-COVID-19-related myocarditis, these inferolateral regional changes were accompanied by frequent involvement of the septum. However, one large multicenter study reported a fairly frequent mild septal LGE in a group of 550 post-COVID-19 patients with protracted symptoms suggestive of cardiac involvement [31]. Left ventricular systolic deformation assessed by 2D strain echocardiography also highly correlated with the amount and region of edema detected by CMR in patients with acute myocarditis. In patients with persistent myocarditis with normal LVEF, global left ventricular longitudinal strain was also reduced, in contrast to results obtained in healthy subjects [32]. Our present results are in line with these findings because the mean left ventricular GLS was reduced and the mean LVEF was within the normal range 

CMR is an imaging modality that can characterize the acute inflammatory myocardial process using a combination of scanning sequences that detect edema, hyperemia, and fibrosis. This combination of sequences represents the foundation of the Lake Louise criteria (LLC) [4]. However, more recent quantitative imaging modalities, such as cardiac T1 and T2 maps, may improve CMR diagnostic accuracy and are currently recommended for clinical use by the expert panel [5]. In addition, another recently introduced technique, feature tracking (FT), may further improve the initial assessment of patients with acute myocarditis. The feature tracking modality is able to analyze segmental and global myocardial strain using routinely obtained cardiac magnetic resonance cine series; however, the usefulness of myocardial strain analysis as an additional diagnostic tool in CMR is not yet well established.

Several studies have investigated whether myocardial strain analysis could discriminate patients with acute myocarditis from healthy subjects and to what extent myocardial strain deformation is associated with other myocardial inflammatory parameters such as prolonged T1 and/or T2. It has also been extensively investigated which type of strain (global, regional, longitudinal, radial, or circumferential) provides the best value in the diagnostic process of patients with acute myocarditis [33,34]. Global peak systolic longitudinal strain (GLS) was shown to be the only strain parameter significantly correlated with global native T1 relaxation time [35].

In our study, GLS was also significantly correlated with T1 relaxation time. Overall, the evidence suggests that longitudinal strain can detect even mild myocardial dysfunction. The newer parameters of myocardial inflammation, especially T1 and T2 relaxation times, have a high diagnostic performance in detecting acute myocardial edema with excellent diagnostic accuracy [11]. The fact that only T1 and not T2 were significantly related to GLS can be because T1 increases can be found in both the acute and chronic phases of myocardial injury, the latter being far more prevalent in our group.

Our results suggest that regional inflammatory processes can lead to regional myocardial dysfunction. Therefore, segmental myocardial strain assessment can be considered a noninvasive parameter of regional myocardial injury in the course of the myocardial inflammatory process. Fisher et al. showed in their univariate analysis that GLS was significantly associated with major adverse cardiovascular events. In a multivariable model adjusted for clinical variables (age, sex, body mass index, and infection intensity parameters) and established CMR parameters (LVEF and LGE amount), LV global longitudinal strain remained independently associated with cardiovascular outcomes [30]. The authors suggested that myocardial strain using feature tracking by CMR has independent prognostic value in addition to clinical data, LVEF, and LGE in patients with myocarditis. Thus, GLS may be a novel marker to improve risk stratification in patients with acute myocarditis. The mean GLS of the entire population in this study was −12.5 ± 4.5%, which indicated a greater degree of impairment compared to our results. This difference could be explained by the fact that the patients in Fisher’s study were referred for CMR with suspected myocarditis and had more severe symptoms and a lower mean LVEF. In contrast to our work, parametric T1 and T2 mapping and extracellular volume were not available in this study. In addition, GLS worse than −13.1% was associated with a higher likelihood of MACE. In our study, left ventricular GLS worse than −15% was associated with a 7.5-fold higher probability of prolonged T1, a well-established marker of myocardial injury and edema. Additionally, the ROC curve suggested that a GLS of −14.5% could predict prolonged T1 relaxation time with the best combination of sensitivity and specificity, but with borderline significance.

To our knowledge, the current study is the first to evaluate FT-derived left ventricular GLS in patients who recently recovered from COVID-19. The CMR-FT used in this study is a technique that has not yet been performed on a routine basis in the clinical setting. However, it may be an important tool for a more detailed characterization of myocardial injury and prognostication in this clinical scenario.

### Limitations

One limitation of this study is that acute myocarditis was not confirmed by endomyocardial biopsy, although biopsy is not a standard diagnostic tool in all cases of suspected acute myocarditis. The strain analysis was also limited to global and segmental longitudinal strains, and no control group without COVID-19 infection was evaluated. Another important consideration is that in patients with cardiac symptoms, CMR provides a noninvasive and clinically useful differential diagnosis across a spectrum of myocardial disease, beyond myocarditis [36]. Therefore, changes in left ventricular deformation are non-specific and can be detected in various cardiac conditions, such as patients with myocardial infarction, left ventricular hypertrophy, amyloidosis, or subclinical coronary artery diseases [37]. It should be underlined that our results cannot be extended to all patients with suspected myocarditis, as only patients with suspected myocarditis after SARS-CoV-2 infection were enrolled, and myocardial strain results should be interpreted in the appropriate clinical context. Additionally, due to the limited sample size of this observational study, the results should be considered hypothesis-generating and need to be validated in a larger cohort with a control group.

## 5. Conclusions

CMR abnormalities, including a myocarditis pattern of nonischemic late gadolinium enhancement, are common findings in patients with prolonged symptoms who have recovered from COVID-19. Changes in CMR global and segmental longitudinal left ventricular strain are related to prolonged T1 relaxation time and may serve as a readily available novel parameter to detect global and regional myocardial injury and dysfunction in patients with suspected myocardial involvement after recovery from COVID-19, thus improving the global diagnostic potential of CMR, including facilities where newer quantitative techniques may not yet be available.

## Figures and Tables

**Table 1:** Study population characteristics.

Age, years	41.8 ± 10.6
Female, n (%)	59 (73)
BMI, kg/m^2^	25.8 ± 4.4
History	
Smoking, n (%)	5 (6.0)
Hyperlipidemia, n (%)	3 (4.0)
Chronic kidney disease, n (%)	1 (1.2)
Hypertension, n (%)	13 (16)
Bronchial asthma, n (%)	5 (6.0)
Hyperthyroidism, n (%)	12 (15)
Clinical presentation	
Respiratory symptoms, n (%)	43 (53)
Chest pain, n (%)	42 (52)
Palpitation, n (%)	47 (58)
Dyspnea, n (%)	28 (35)
Initial blood testing	
Troponin I, ng/mL	0.003 ± 0.001
Hemoglobin, g/dL	13.8 ± 1.3
CRP, mg/L	3.2 ± 5.2
Hematocrit, (%)	40.4 ± 3.6
Glucose, mg/dL	94.3 ± 10.1
LVEF, (%)	59.4 ± 5.9

LVEF—left ventricle ejection fraction.

**Table 2:** CMR LV myocardial parameters in patients with and without significantly reduced GLS.

	GLS More Negative or Equal −15 n = 53	GLS Less Negative than −15 n = 28	*p*
T1(ms), mean ± SD	1004.1 ± 23.0	1007.7 ± 37.0	0.642
T1 > norm, (>1035 ms)	4 (7.5)	8 (28.6)	0.019
T2 (ms), mean ± SD	47.5 ± 1.8	47.6 ± 3.8	0.898
T2 > norm, (>49 ms)	14 (26.4)	7 (25.0)	0.89
ECV (%), mean ± SD	27.0 ± 2.2	28.3 ± 8.9	0.519
ECV > norm, (>29%)	7 (13.2)	6 (21.4)	0.356
LVEF (%), mean ± SD	61.6 ± 4.8	53.6 ± 8.9	<0.001
LGE (+)	30 (56.6)	22 (78.6)	0.05

GLS—global longitudinal strain; ECV—extracellular volume fraction; LVEF—left ventricular ejection fraction; LGE—late gadolinium enhancement.

**Table 3:** Correlation of GLS with T1, T2, ECV, and LVEF.

	GLS (%)	
	r	*p*
T1 (ms)	0.303	0.006
T2 (ms)	0.193	0.084
ECV (%)	−0.049	0.667
LVEF (%)	−0.732	<0.001

GLS—global longitudinal strain; ECV—extracellular volume fraction; LVEF—left ventricular ejection fraction. T1- global longitudinal relaxation time of the myocardium, measured as a mean value of the global T1 in basal and midventricular slices.

**Table 4:** Multivariate logistic regression analysis for impaired global longitudinal strain (less negative than −15%).

GLS Less Negative than −15%	Univariate Logistic Regression	Multivariate Logistic Regression		
	*p*	*p*	HR	95% CI
High global T1 (ms)	0.017	0.028	7.62	1.25–46.64
High global T2 (ms)	0.89	0.313	0.51	0.14–1.88
High global ECV (%)	0.342	0.584	0.61	0.10–3.66
LGE (+)	0.054	0.079	2.69	0.89–8.16

GLS—global longitudinal strain, ECV—extracellular volume fraction, LGE—late gadolinium enhancement.

**Table 5:** Univariate and multivariate logistic regression of SLS, F1, T2, and ECV was used to evaluate the effect of impaired left ventricular GLS less negative than −15%.

GLS Less Negative than −15 Continuous Variables	Univariate Logistic Regression	Multivariate Logistic Regression		
	*p*	*p*	HR	95% CI
SLS seg 5	0.001	0.039	1.66	1.03–2.68
T1 seg 5	0.016	0.748	1.01	0.96–1.06
T2 seg 5	0.423			
ECV seg 5	0.268			
SLS seg 6	0.01	0.279	1.17	0.88–1.57
T1 seg 6	0.009	0.409	1.03	0.97–1.09
T2 seg 6	0.655			
ECV seg 6	0.123			
SLS seg 10	0.002	0.952	0.99	0.71–1.39
T1 seg 10	0.089	0.371	0.98	0.93–1.03
T2 seg 10	0.782			
ECV seg 10	0.232			

SLS: segmental longitudinal strain; GLS: global longitudinal strain; ECV: extracellular volume fraction.

## Data Availability

Data are available from the corresponding author upon reasonable request.
